# Emerging roles of circular RNAs in neuropathic pain

**DOI:** 10.1111/cpr.13139

**Published:** 2021-10-08

**Authors:** Derong Xu, Xuexiao Ma, Chong Sun, Jialuo Han, Chuanli Zhou, Matthew T. V. Chan, William K. K. Wu

**Affiliations:** ^1^ Department of Spine Surgery The Affiliated Hospital of Qingdao University Qingdao Shandong China; ^2^ Department of Anaesthesia and Intensive Care and Peter Hung Pain Research Institute The Chinese University of Hong Kong Hong Kong China; ^3^ State Key Laboratory of Digestive Diseases LKS Institute of Health Sciences The Chinese University of Hong Kong Hong Kong China

**Keywords:** circAnks1a, circHIPK3, circRNAs, circular RNAs, neuropathic pain

## Abstract

Neuropathic pain is a major type of chronic pain caused by the disease or injury of the somatosensory nervous system. It afflicts about 10% of the general population with a significant proportion of patients’ refractory to conventional medical treatment. This highlights the importance of a better understanding of the molecular pathogenesis of neuropathic pain so as to drive the development of novel mechanism‐driven therapy. Circular RNAs (circRNAs) are a type of non‐coding, regulatory RNAs that exhibit tissue‐ and disease‐specific expression. An increasing number of studies reported that circRNAs may play pivotal roles in the development of neuropathic pain. In this review, we first summarize circRNA expression profiling studies on neuropathic pain. We also highlight the molecular mechanisms of specific circRNAs (circHIPK3, circAnks1a, ciRS‐7, cZRANB1, circZNF609 and circ_0005075) that play key functional roles in the pathogenesis of neuropathic pain and discuss their potential diagnostic, prognostic, and therapeutic utilization in the clinical management of neuropathic pain.

## Graphical abstract

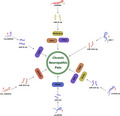

## 1 INTRODUCTION

Neuropathic pain is a chronic pain condition caused by disease or injury of the somatosensory nervous system.^1^, ^2^, ^3^, ^4^, ^5^ It encompasses aetiologically distinct yet mechanistically similar disease entities, including postherpetic neuralgia, trigeminal neuralgia, painful diabetic neuropathy, cancer‐related and chemotherapy‐induced neuropathic pain, and neural injury or impingement, such as spinal cord injury and nerve root compression.^6^, ^7^, ^8^, ^9^, ^10^, ^11^ Although the exact prevalence of neuropathic pain varies from country to country, it has been estimated that up to 10% of the general population is afflicted with this potentially disabling condition.^12^, ^13^, ^14^, ^15^, ^16^ Different medical treatments (eg tricyclic antidepressants, selective serotonin noradrenaline reuptake inhibitors, opioids, lidocaine) have been used clinically for the treatment of neuropathic pain, but up to half of the patients with neuropathic pain are refectory to these drugs.^17^, ^18^, ^19^, ^20^, ^21^, ^22^ The ineffectiveness of medical interventions arises partly due to the poorly understood molecular mechanism of neuropathic pain. Both peripheral and central sensitization are known to be implicated in the pathogenesis of neuropathic pain.^23^, ^24^, ^25^ Whereas the anomalous excitability of the primary sensory neurons during peripheral sensitization may be due to maladaptive changes in the gene transcription and translation of enzymes, receptors, and voltage‐dependent ion channels in the dorsal root ganglion, neuroinflammation caused by pathological microglia activation takes a major part in the process of central sensitization.^26^, ^27^, ^28^, ^29^ However, how the deranged molecular pathways underlying peripheral and central sensitization could be targeted therapeutically is still an active area of investigation.

Circular RNAs (circRNAs) are a type of non‐coding, regulatory RNAs evolutionarily conserved across mammalian species.^30^, ^31^, ^32^, ^33^, ^34^ CircRNAs exhibit brain region‐specific expression,^35^, ^36^ and the abundance of circRNAs in the brains of various species are largely similar.^37^, ^38^, ^39^, ^40^ CircRNAs exert their biological functions principally by acting as competing endogenous RNAs (ceRNAs) to regulate gene expression post‐transcriptionally by sponging microRNAs (miRNAs).^41^, ^42^, ^43^, ^44^ CircRNAs have been shown to be deregulated in different human diseases, including neurological disorders.^45^, ^46^ Recently, studies found that circRNAs may play important roles in the development of neuropathic pain.^47^, ^48^

In our review, we firstly summarize circRNA expression profiling studies on neuropathic pain so as to provide the scientific community with a comprehensive collection of data sets for subsequent integrative analysis. The biological functions and molecular mechanisms of specific circRNAs involved in the pathogenesis of neuropathic pain are also discussed in relation to their diagnostic, prognostic and therapeutic potentials in clinical settings.

## 2 CIRCRNA EXPRESSION PROFILING AND INTEGRATIVE ANALYSIS IN NEUROPATHIC PAIN

Microarray is an efficient tool for circRNA profiling. Cao and colleagues inflicted chronic constriction injury (CCI) to the sciatic nerve of rats to induce neuropathic pain.^49^ They then extracted total RNA from ipsilateral spinal dorsal horns of CCI and sham‐operated rats and performed circRNA microarray to analyse circRNA expression patterns. They found that there were 469 differentially expressed circRNAs (363 upregulated and 106 downregulated) in the CCI group compared to the sham‐operated group. The expression levels of three circRNAs (circRNA_003724, circRNA_008008 and circRNA_013779) were increased by more than 10 folds after CCI. Furthermore, reverse transcription‐quantitative PCR (RT‐qPCR) was used to confirm the deregulation of circRNA_008973, circRNA_013779, circRNA_008646, circRNA_35215, circRNA_011111, circRNA_007419, circRNA_007512 and circRNA_010913. CeRNAs network reconstruction indicated that circRNA_013779 and circRNA_008008 are the two key nodes in the circRNA‐miRNA interaction network amongst the top 10 differentially expressed circRNAs. Cao and colleagues also performed the circRNA microarray to identify differentially expressed circRNAs in the postherpetic neuralgia (PHN) skin and the control skin.^50^ They showed that only circRNA_405463 showed differential expression when fold change cut‐off was set as ≥2.0 between the PHN and control group. With a less stringent cut‐off (fold change ≥1.5), the number of downregulated and upregulated circRNAs increased to 8 and 23, respectively. They also performed miRNA microarray on the same set of samples, which identified of 317 differently expressed miRNAs in the PHN skin (fold change ≥2.0). To learn the functions of these differential miRNAs, their potential target mRNAs were predicted and analysed by Genomes pathway, Kyoto Encyclopaedia of Genes (KEGG) and Gene Ontology (GO) enrichment analysis. Target mRNAs were found to be enriched in pathways such as AMP‐activated protein kinase (AMPK), mitogen‐activated protein kinase (MAPK) and forkhead box O (FoxO) signalling.

With the advancement of linear RNA depletion and bioinformatic workflow, circRNAs have been increasingly profiled by RNA sequencing. Zhou and colleagues performed RNA sequencing to profile the expression levels of non‐coding RNAs (ncRNAs) in the spinal cord after spared nerve injury (SNI)‐induced neuropathic pain.^48^ They showed that 188 circRNAs were differentially expressed (68 upregulated and 120 downregulated) in the rat spinal cord on day 14 after SNI as compared to the control group. There were also 144 differentially expressed lncRNAs (15 upregulated and 129 downregulated) and 12 differentially expressed miRNAs (6 upregulated and 6 downregulated), and 1066 differentially expressed mRNAs (531 upregulated and 535 downregulated) in the rat spinal cord after SNI at the same time point, in which circ_0006928‐miR‐184 and LNC_001457‐miR‐184 interactions were verified to play a crucial role in excessive neuronal cell apoptosis in the spinal cord after SNI. Zhang and colleagues also performed RNA sequencing to profile circRNA expression in the rat spinal dorsal horn on day 7 and day 14 after spinal nerve ligation (SNL).^51^ They identified a total of 61,833 distinct circRNAs according to the criterion of at the least one back spliced junction reads. Amongst them, the reads per million mapped reads (RPM) of 12,849 circRNAs was greater than 0.1. However, only 21 circRNAs were identified to be significantly deregulated with >2.5‐fold change at both time points.

**FIGURE 1:**
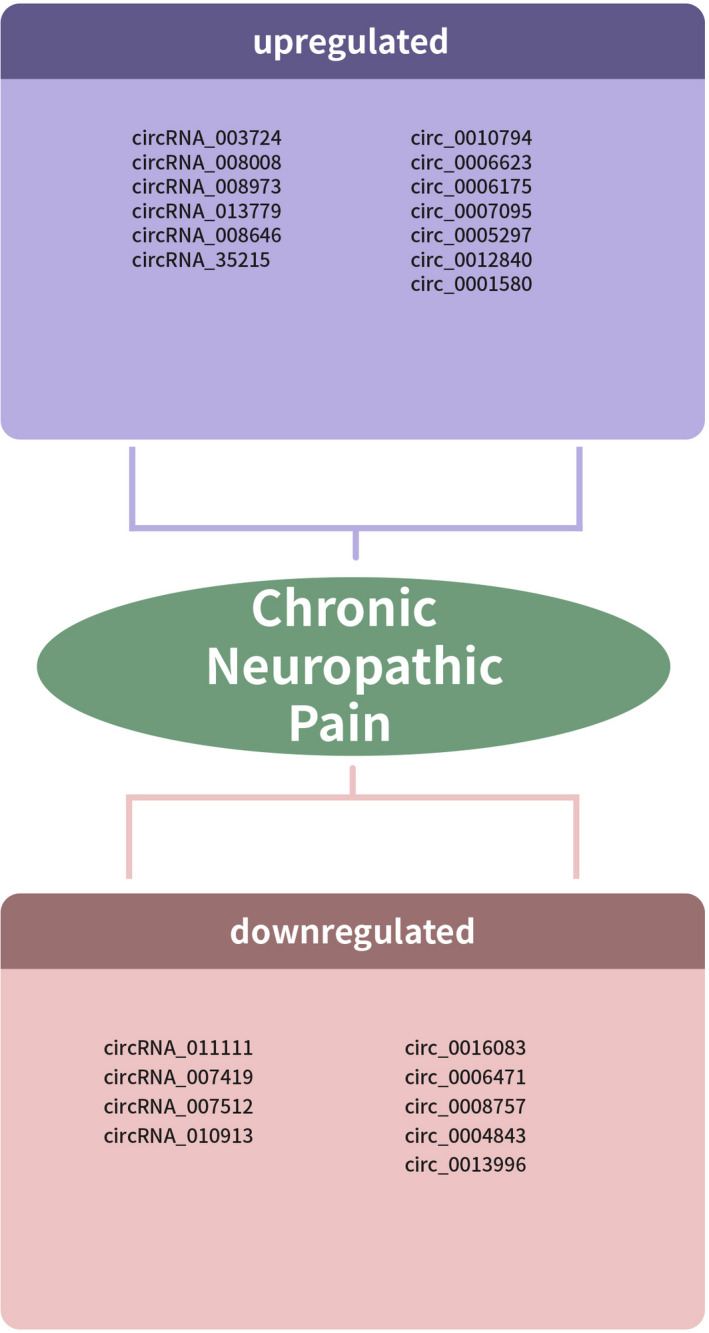
Dysregulated circRNAs in neuropathic pain

Recently, He et al. performed RNA sequencing to study the expression patterns of circRNAs, lncRNAs and miRNAs in the spinal cord of streptozotocin‐induced diabetic neuropathic pain (DNP) mice.^52^ They found that there were 135 circRNAs were differentially expressed and 71 circRNAs were downregulated and 64 circRNAs were overexpressed in spinal cord between control group and DNP group. Amongst these, circ_0010794, circ_0006623, circ_0006175, circ_0007095, circ_0005297, circ_0012840 and circ_0001580 was overexpressed and circ_0016083, circ_0006471, circ_0008757, circ_0004843 and circ_0013996 was downregulated (Figure 1 and Table 1).

**TABLE 1:** CircRNAs expression profiles in neuropathic pain

Num	Method	Sample	Upregulated	Downregulated	References
1	Microarray PCR	Ipsilateral spinal dorsal horns	363 circRNAs circRNA_003724, circRNA_008008 circRNA_008973 circRNA_013779 circRNA_008646, circRNA_35215	106 circRNAs circRNA_011111, circRNA_007419, circRNA_007512 and circRNA_010913	^49^
2	Microarray	Postherpetic neuralgia skin	66 CircRNAs	68 CircRNAs	^50^
3	RNA sequencing	Spinal cord	68 circRNAs	120 circRNAs	^48^
4	RNA sequencing	Spinal dorsal horn	54 circRNAs	52 circRNAs	^51^
5	RNA sequencing	Spinal cord	64 circRNAs circ_0010794, circ_0006623, circ_0006175, circ_0007095, circ_0005297, circ_0012840 circ_0001580	71 circRNAs circ_0016083, circ_0006471, circ_0008757, circ_0004843 circ_0013996	^52^

## 3 FUNCTIONS AND MECHANISMS OF ACTION OF NEWLY DISCOVERED CIRCRNAS IN NEUROPATHIC PAIN

### 3.1 CircHIPK3

CircHIPK3 is a circRNA that has been shown to function as a tumour suppressor gene or oncogene in a context‐dependent manner to modulate tumour progression through sponging miRNAs.^53^, ^54^, ^55^ Wang and colleagues investigated the potential regulatory role of circHIPK3 in the development of diabetic neuropathic pain.^56^ Their data showed that circHIPK3 abundance was increased in the dorsal root ganglion from streptococci‐induced diabetic rats and serum from patients with diabetic neuropathic pain. Higher expression of circHIPK3 was positively correlated with the grade neuropathic pain in cases with type 2 diabetes. Functionally, knockdown of circHIPK3 alleviated neuropathic pain in the diabetic rats and suppressed interleukin (IL)‐12, tumour necrosis factor (TNF)‐α, IL‐1β and IL‐6. Moreover, they showed that circHIPK3 was found to sponge miR‐124 to contribute to neuroinflammation and exacerbate neuropathic pain in the diabetic rats. Therefore, circHIPK3 may be a potential therapeutic target for the treatment of neuropathic pain.

### 3.2 CircAnks1a

Zhang and colleagues identified the aberrant upregulation of circAnks1a in the rat spinal dorsal horn after SNL by RNA sequencing.^51^ CircAnks1a was found to be localized in both the nucleus and cytoplasm. Knockdown of circAnks1a attenuated the pain‐like behaviour caused by SNL. Mechanistically, circAnks1a increased the interaction between transportin‐1 and transcription factor YBX1 and thereby inducing the nuclear translocation of YBX1 from the cytoplasm. CircAnks1a also directly bind to Vegfb promoter and promoted YBX1 recruitment to facilitate transcription of *Vegfb*. Moreover, cytoplasmic circAnks1a acted as a miRNA sponge to repress miR‐324‐3p to disinhibit VEGFB. Overexpression of VEGFB contributed to the increased excitability of the dorsal horn neurons and SNL‐induced pain. These data suggested that the circAnks1a‐miR‐324‐3p‐VEGFB axis is a novel therapeutic target in neuropathic pain.

### 3.3 ciRS‐7

The circRNA ciRS‐7 has been found to take part in the development of different diseases.^57^, ^58^, ^59^, ^60^ For example, Han and colleagues demonstrated that ciRS‐7 induced migration and growth through modulating the miR‐7‐EGFR axis in papillary thyroid cancer.^61^ Zhang and colleagues also showed that ciRS‐7 enhanced epithelial‐mesenchymal transition through sponging miR‐641 to derepress MDM2 and ZEB1 expression.^62^ In the CCI model of neuropathic pain, Cai et al. found that ciRS‐7 expression in the rat spinal cord dorsal horn was positively correlated with development of neuropathic pain partly through promoting inflammation, in which knockdown of ciRS‐7 attenuated microglia activation and expression of pro‐inflammatory cytokines IL‐6, IL‐12 and TNFα.^63^ Mechanistically, ciRS‐7 bound to and increased the availability of miR‐135a‐5p, whose inhibition also mitigated neuroinflammation and neuropathic pain. Their data indicated that either targeting ciRS‐7 or miR‐135a‐5p could alleviate neuropathic pain through suppressing neuroinflammation.

### 3.4 cZRANB1

Wei and colleagues studied the expression and functional role of miR‐24‐3p in the development of neuropathic pain in the CCI rat models.^64^ They found that miR‐24‐3p expression was upregulated in the dorsal spinal cords of CCI rats, in which ablation of miR‐24‐3p significantly alleviated thermal hyperalgesia and mechanical allodynia. Moreover, miR‐24‐3p upregulated Wnt5a‐β‐catenin signalling pathway to induce neuropathic pain by inhibiting LPAR3 expression. As the upstream modulator, the circRNA cZRANB1 was found to sponge miR‐24‐3p as predicted by bioinformatics analysis and confirmed by luciferase reporter assay and biotinylated RNA pull‐down. Importantly, cZRANB1 expression was decreased in CCI rats, in which enforced expression of cZRANB1 alleviated thermal hyperalgesia and mechanical allodynia. The regulation of miR‐24‐3p‐LPAR3 axis by cZRANB1 was also confirmed in the CCI model.

### 3.5 CircZNF609

Li and colleagues demonstrated that the expression of miR‐22‐3p was downregulated in the dorsal spinal cord of CCI rats at the postoperative day 0, 3, 7, 10 and 14 as compared to the sham‐operated rats.^65^ Enforced expression of miR‐22‐3p attenuated neuropathic pain and suppressed the expression of pro‐inflammatory cytokines IL‐6, TNF‐α and IL‐1. Moreover, ENO1 was identified to be a direct target gene for miR‐22‐3p. Downregulation of miR‐22‐3p alleviated the thermal hyperalgesia and mechanical allodynia partly through targeting ENO1 expression. They also showed that the circRNA circZNF609, which was upregulated in CCI rats, was a sponge for miR‐22‐3p. Functionally, knockdown of circZNF609 alleviated thermal hyperalgesia and mechanical allodynia levels and suppressed IL‐6, TNF‐α and IL‐1 expression by regulating miR‐22‐3p‐ENO1 axis. These data suggested that circZNF609 induced inflammation factors to mediate central sensitization in neuropathic pain development through regulating miR‐22‐3p‐ENO1 axis.

### 3.6 Circ_0005075

**FIGURE 2:**
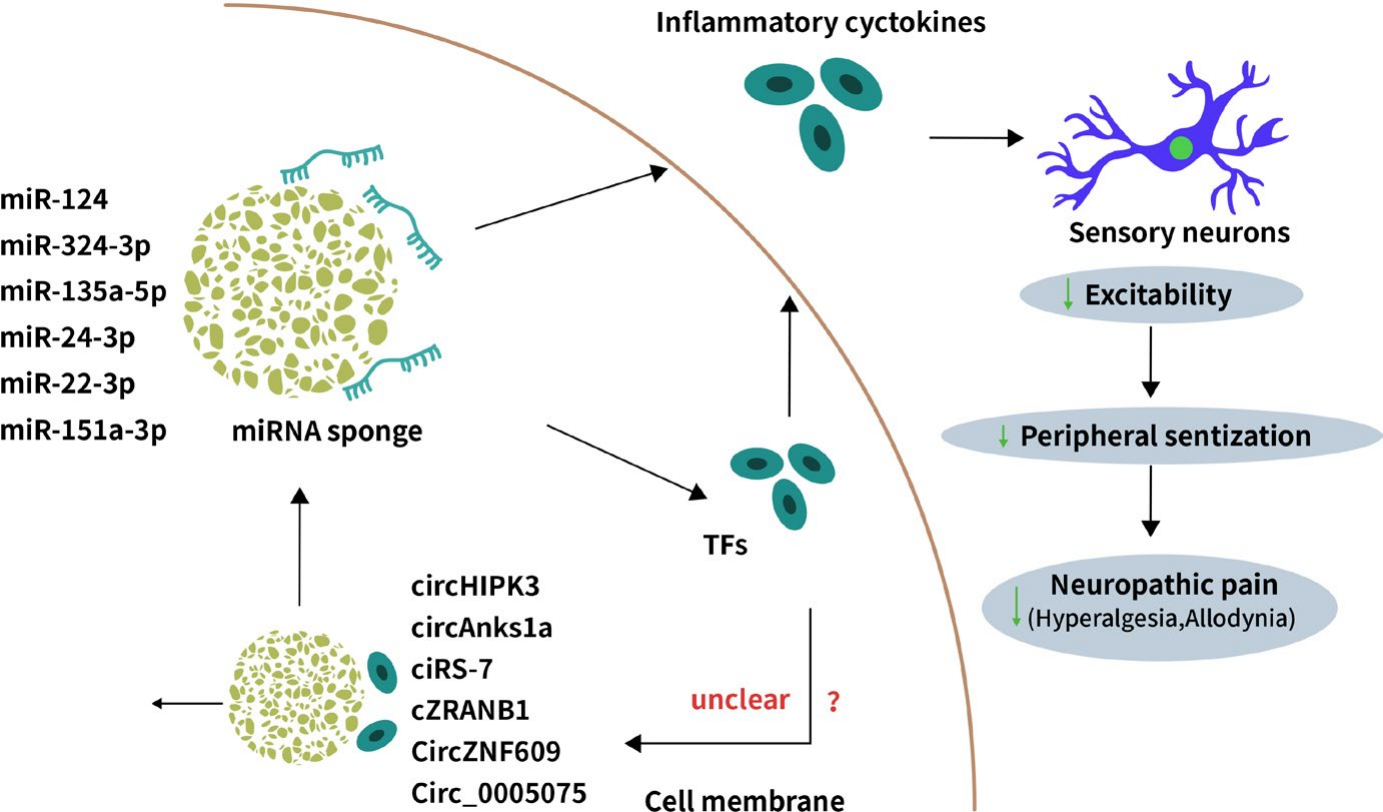
Schematic of circRNA‐miRNA interactions in neuropathic pain. circRNAs functions as ‘miRNA sponge’ to reduce the expression levels of miRNA in NP, it prevents TFs (such as TNFAIP1, ZEB1, STAT3) from microRNA‐mediated suppression, or directly decrease the release of inflammatory cytokines, thus alleviating the symptoms of neuropathic pain

**FIGURE 3:**
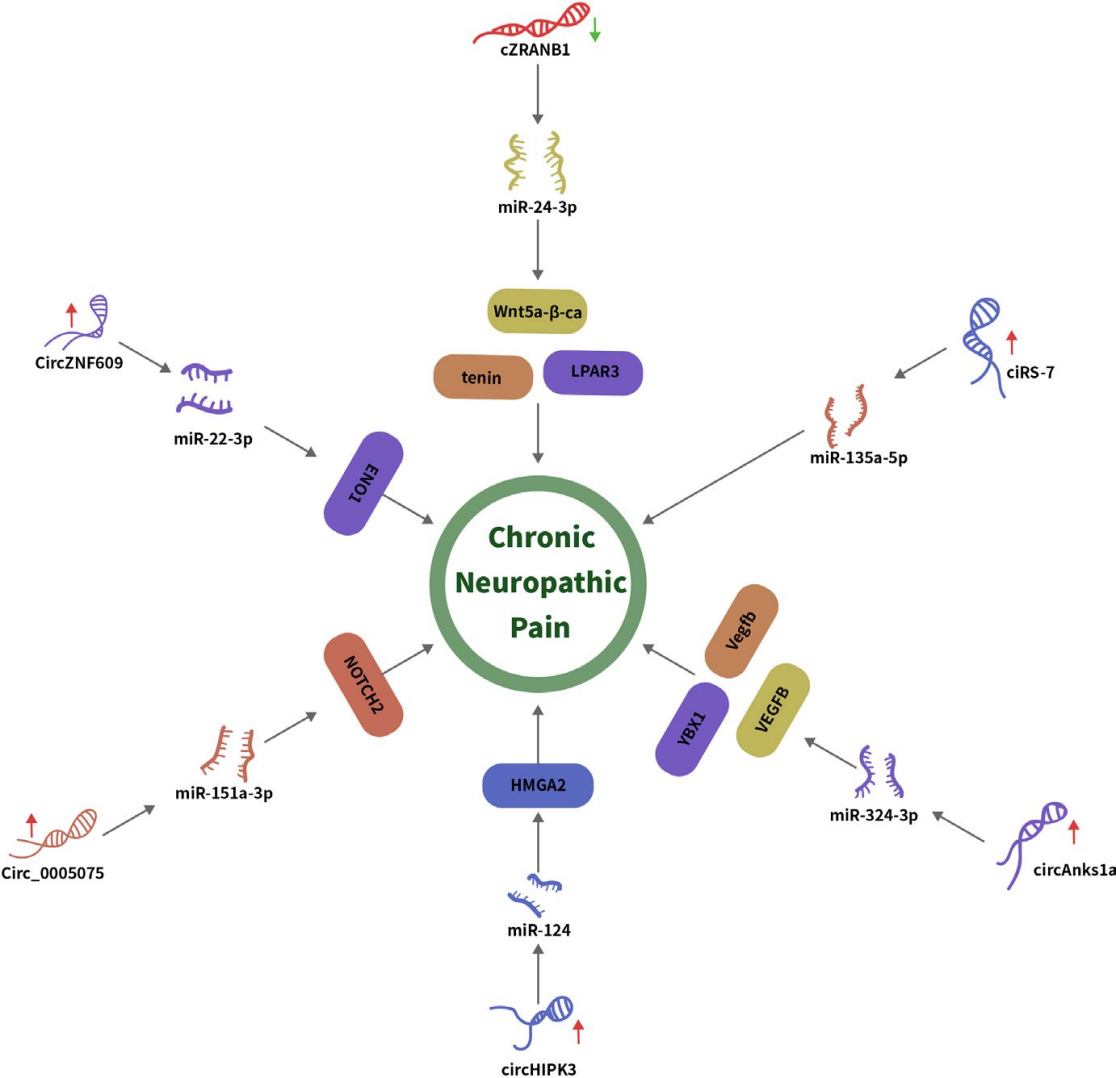
CircRNAs regulated genes expression via sponging miRNAs and played crucial roles in excitability and inflammation in neuropathic pain

circ_0005075 deregulation has been implicated in cancer development.^66^, ^67^, ^68^, ^69^, ^70^ Zhang and colleagues showed that circ_0005075 was upregulated in the dorsal spinal cord of CCI rats, in which knockdown of circ_000507 suppressed neuropathic pain behaviours such as thermal hyperalgesia and mechanical allodynia.^47^ Knockdown of circ_0005075 also inhibited neuroinflammation through targeting TNF‐α, IL‐6, IL‐10 and cyclooxygenase (COX)‐2. Mechanistically, circ_0005075 was found to sponge miR‐151a‐3p and derepress NOTCH2 to mediate its promoting effects on neuroinflammation and neuropathic pain development (Figures 2, 3 and Table 2).

**TABLE 2:** Dysregulated circRNAs in neuropathic pain.

Name	Dysregulation	Sponge target	Function	Related gene	Role	References
circHIPK3	Upregulated	miR‐124	Neuroinflammation	HMGA2	Harmfulness	^56^
circAnks1a	Upregulated	miR‐324‐3p	Excitability inflammation	YBX1 Vegfb VEGFB	Harmfulness	^51^
ciRS‐7	Upregulated	miR‐135a‐5p	Inflammation		Harmfulness	^63^
cZRANB1	Downregulated	miR‐24‐3p	Inflammation	Wnt5a‐β‐catenin LPAR3	Protective	^64^
CircZNF609	Upregulated	miR‐22‐3p	Inflammation	ENO1	Harmfulness	^65^
Circ_0005075	Upregulated	miR‐151a‐3p	Neuroinflammation	NOTCH2	Harmfulness	^47^

## 4 CONCLUSIONS AND FUTURE PERSPECTIVES

Neuropathic pain is a serious public health issue that is poorly tackled by medical treatment, representing an unmet medical need. Emerging molecular studies have shed new light on the mechanisms of peripheral and central sensitization in neuropathic pain.

The increasing number of studies have suggested that circRNAs play crucial roles in the development of neuropathic pain through neuroinflammation in both the dorsal root ganglia and spinal cord dorsal horns. From the mechanistic point of view, circRNAs may regulate glial activation and expression of the pro‐inflammatory genes by sponging pain‐related miRNAs (miR‐124, miR‐324‐3p, miR‐135a‐5p, miR‐24‐3p, miR‐22‐3p and miR‐151a‐3p). These studies have also supported the potential clinical utility of circRNAs and their downstream signalling mediators as therapeutic targets. In this connection, different approaches could be used to target pain‐related circRNAs—(1) CRISPR/Cas9‐mediated ablation; (2) antisense oligonucleotides or small interfering RNAs‐mediated knockdown; and (3) steric blockade of circRNA‐miRNA interactions by morpholinos. Through circRNA microarray and RNA sequencing, a growing number of deregulated circRNAs are expected to be identified in neuropathic pain. The potential implications in the clinical diagnosis and prognostication of circRNAs in neuropathic pain will be achieved. Then, it needs to measure these deregulated circRNAs in large samples of neuropathic pain. However, unlike other ncRNAs, such as long non‐coding RNAs (lncRNA), the correlation between pain scores of patients and circRNA levels in the plasma has not yet to be demonstrated. Further research in this direction will help identify novel biomarkers for monitoring patients with neuropathic pain. However, more in‐depth functional and mechanistic investigations on pain‐related circRNAs are warranted. It is hopeful that, with more translational studies, circRNAs will one day be utilized for the clinical management of patients with neuropathic pain.

## CONFLICT OF INTERESTS

The authors declare that they have no competing interests.

## AUTHOR CONTRIBUTIONS

DRX, XXM, CS, JL H, CLZ: drafted and wrote the manuscript. MTVC and WKKW: revised the manuscript. DRX and CLZ: participated in the design of the review. All authors read and approved the final manuscript.

## Data Availability

Research data are not shared.
